# Maintenance Chemotherapy for Patients with Rhabdomyosarcoma

**DOI:** 10.3390/cancers15154012

**Published:** 2023-08-07

**Authors:** Gianni Bisogno, Veronique Minard-Colin, Meriel. Jenney, Andrea Ferrari, Julia Chisholm, Daniela Di Carlo, Lisa Lyngsie Hjalgrim, Daniel Orbach, Johannes Hendrikus Maria Merks, Michela Casanova

**Affiliations:** 1 Department of Women’s and Children’s Health, University of Padua, 35128 Padua, Italy; danieladicarlo25@gmail.com; 2 Pediatric Hematology Oncology Division, University Hospital of Padua, 35128 Padua, Italy; 3 Department of Pediatric and Adolescent Oncology, Institut Gustave-Roussy, Université Paris-Saclay, 94800 Villejuif, France; veronique.minard@gustaveroussy.fr; 4 Department of Paediatric Oncology, Children’s Hospital for Wales, Heath Park, Cardiff CF14 4XW, UK; meriel.jenney@wales.nhs.uk; 5 Pediatric Oncology Unit, Fondazione IRCCS Istituto Nazionale Tumori, 20133 Milan, Italy; andrea.ferrari@istitutotumori.mi.it (A.F.); michela.casanova@istitutotumori.mi.it (M.C.); 6 Children and Young People’s Unit, Royal Marsden Hospital and Institute of Cancer Research, Sutton SM2 5PT, UK; julia.chisholm@rmh.nhs.uk; 7 Department of Paediatric and Adolescent Medicine, University Hospital Copenhagen, 2100 Copenhagen, Denmark; lisa.lyngsie.hjalgrim@regionh.dk; 8 SIREDO Oncology Centre (Care, Innovation and Research for Children, Adolescents and Young Adults with Cancer), Institut Curie, Paris Sciences et Lettres L University, 75005 Paris, France; daniel.orbach@curie.fr; 9 Princess Máxima Centre for Pediatric Oncology, 3584 CS Utrecht, The Netherlands; j.h.m.merks@prinsesmaximacentrum.nl; 10 Division of Imaging and Oncology, University Medical Center Utrecht, 3584 CX Utrecht, The Netherlands

**Keywords:** maintenance chemotherapy, metronomic chemotherapy, low-dose chemotherapy, rhabdomyosarcoma

## Abstract

**Simple Summary:**

The updated results of the RMS2005 randomized study confirm that patients with non-metastatic high risk rhabdomyosarcoma have an improved survival when maintenance chemotherapy (MC) with vinorelbine and low dose cyclophosphamide is added to the standard multidisciplinary treatment. A more recent randomized study adopted the same strategy, but different drugs were used in the MC phase (trofosfamide, idarubicin and etoposide). No survival improvement was evident in the MC group, suggesting that not all types of MC are equally effective. A revision of the literature demonstrates that the role of MC in patients with metastatic or relapsed RMS may be a promising approach but need more investigations.

**Abstract:**

Maintenance chemotherapy (MC) defines the administration of prolonged relatively low-intensity chemotherapy with the aim of “maintaining” tumor complete remission. This paper aims to report an update of the RMS2005 trial, which demonstrated better survival for patients with high-risk localized rhabdomyosarcoma (RMS) when MC with vinorelbine and low-dose cyclophosphamide was added to standard chemotherapy, and to discuss the published experience on MC in RMS. In the RMS2005 study, the outcome for patients receiving MC vs. those who stopped the treatment remains superior, with a 5-year disease-free survival of 78.1% vs. 70.1% (*p* = 0.056) and overall survival of 85.0% vs. 72.4% (*p* = 0.008), respectively. We found seven papers describing MC in RMS, but only one randomized trial that did not demonstrate any advantage when MC with eight courses of trofosfamide/idarubicine alternating with trofosfamide/etoposide has been employed in high-risk RMS. The use of MC showed better results in comparison to high-dose chemotherapy in non-randomized studies, including metastatic patients, and demonstrated feasibility and tolerability in relapsed RMS. Many aspects of MC in RMS need to be investigated, including the best drug combination and the optimal duration. The ongoing EpSSG trial will try to answer some of these questions.

## 1. Introduction

Maintenance therapy in oncology is commonly intended as a treatment given after the tumor has disappeared (i.e., achieving complete remission) after initial induction therapy, aiming to treat potential minimal residual disease [1]. It generally includes the administration of prolonged chemotherapy, but other agents, such as antibodies and/or differentiating or targeted drugs can be employed [2,3].

The type and length of the maintenance phase varies considerably, but generally, it is less intensive than the induction treatment in order to allow a day care administration. When anticancer agents are provided at very low doses, MC includes a metronomic approach, and therefore alternative mechanisms of action are hypothesized for the drugs used [4]. 

Maintenance chemotherapy (MC) is a well-established treatment strategy for patients with acute lymphoblastic leukemia [5]. There is less experience in pediatric solid tumors, with some evidence of improved results in neuroblastoma but not in osteosarcoma [4,6,7].

The concept of MC has garnered new interest after the results of the European paediatric Soft tissue sarcoma Study Group (EpSSG) RMS 2005 trial were published. This randomized trial demonstrated better survival for patients with high-risk localized rhabdomyosarcoma (RMS) when MC with vinorelbine and low-dose cyclophosphamide was administered after standard induction chemotherapy [8].

In this paper, we present an update of the RMS2005 trial data and discuss the recent literature focusing on the role of MC in RMS treatment.

## 2. The Rationale for Maintenance in RMS

Several clinical trials have been performed since the late 1970s which sought to identify the most effective chemotherapy regimen for patients with RMS [9]. In these trials, the multidisciplinary treatment, including chemotherapy, surgery, and/or radiotherapy, causes the complete radiological tumor disappearance in nearly all patients. However, approximately one-third of patients with localized RMS experienced a relapse after a relatively short time, with 2/3 of them presenting an event within one year from the end of treatment [10]. This suggests that radiologically undetectable residual disease may remain at the end of standard treatment and explain why some additional treatment may be of benefit. Conversely, most patients do not relapse after standard treatment, and for them, a prolonged treatment schedule would mean administering unnecessary therapy, thereby increasing the risk of acute and late effects. MC may be the solution to this dilemma, providing that the choice of a chemotherapy regimen only adds limited acute toxicity that, theoretically, may also have different mechanisms of action. MC may also provide the administration of drugs not previously used, or that may overcome a possible drug resistance developed during standard treatment, or the use of a metronomic strategy, defined by the administration of chemotherapeutic drugs at low doses given on a continuous or frequent schedule, over a long period of time, possibly without or with minimal drug-free intervals [4].

## 3. The EpSSG Studies

Before starting the RMS 2005 study, the effectiveness and tolerability of vinorelbine as a single agent and its combination with low-dose cyclophosphamide were proven in relapsed RMS patients [11,12]. The attractiveness of this combination relied on the fact that neither of the two drugs was part of the initial standard regimen based on the administration of nine cycles of ifosfamide, vincristine, and actinomycin-D (IVA), with or without doxorubicin. This could, in theory, overcome the problem of a chemo-resistance that mat have arisen during standard therapy. A phase II prospective study confirmed their efficacy in a relapse situation with an overall response rate of 36% in 50 patients with RMS [13]. 

Cyclophosphamide has always been the cornerstone of the chemotherapy regimens for RMS adopted by the Children’s Oncology Group (COG) Soft-Tissue Sarcoma Committee, and continuous low doses (2.5 mg/kg/day for up to 2 years) were administered in the initial North American studies [14]. When the RMS 2005 trial was initiated, there were suggestions that vinca alkaloids and continuous low-dose cyclophosphamide might also have an anti-angiogenic and immunomodulatory mechanism of action [15,16,17]. These findings provided the rationale for EpSSG to test this combination as MC. 

The RMS 2005 trial was a multicenter, open label, randomized, controlled, phase 3 trial involving 14 different countries and 102 pediatric oncology centers. It included patients 6 months to 21 years old with a histologically proven diagnosis of high-risk non-metastatic RMS. These patients were eligible for two randomized trials: (a) the first comparing IVA (ifosfamide, vincristine, actinomycin-D) vs. IVADo (IVA plus doxorubicin) in the initial part of standard treatment, and [18] (b) the second for the MC randomization [8].

All participating centers to enroll patients in the RMS2005 trial were required to obtain written approval from their local authorities and ethical committees, and written informed consent from patients and/or their parents or legal guardians. The RMS2005 trial is registered with EUDRACT Number 2005-000217-35 and ClinicalTrials.gov number NCT00339118Patients included in the high-risk group were those with a non-metastatic RMS and (a) incompletely resected embryonal RMS with unfavorable features (tumor site, patient age ≥ 10 years and/or tumor size > 5 cm), (b) embryonal RMS with locoregional nodal involvement, and (c) alveolar RMS without locoregional nodal involvement. This high-risk group comprises approximately 50% of patients with RMS.

Standard treatment included the administration of 9 cycles of IVA +/− doxorubicin, surgery, and/or radiotherapy. For the MC trial, specific eligibility criteria were (a) patients with tumor in clinical remission (or with minimal radiological abnormalities on imaging studies) at the end of standard treatment and (b) no severe vincristine-related neuropathy. 

Patients were randomly assigned to stop treatment (standard arm) or receive MC (experimental arm) with six cycles of i.v. vinorelbine 25 mg/m^2^ on days 1, 8, and 15, and daily oral cyclophosphamide 25 mg/m^2^, on days 1 to 28. The total duration of MC was 24 weeks.

The primary endpoint was disease-free survival (DFS), defined as the time from randomization to tumor relapse or death due to any cause at the time of the latest follow-up. Secondary outcomes were overall survival (OS), defined as the time from randomization to death due to any cause, or time to the latest follow-up, and toxicity, assessed according to NCI-CTC version 3. Survival probabilities were estimated according to the intention-to-treat principle, i.e., including patients in the group to which they were assigned, whether they received the allocated treatment or not, using the Kaplan–Meier method and the log-rank test. Median follow-up time was reported for alive patients. 

In this report, we present the RMS 2005 results, updated in March 2022 when the latest analysis was performed.

A total of 371 patients were randomized: 186 (50.1%) in the standard arm and 185 (49.9%) in the experimental arm. One patient received MC despite being randomized to stop treatment and three patients assigned to the experimental arm did not receive MC. The two groups were similar regarding clinical characteristics and treatment received before entering the randomized trial. Treatment was completed as per protocol in 165 out of 183 patients who started MC (including the one randomized to stop the treatment). The most common adverse event was grade 4 neutropenia, which occurred in 45% of patients, and grade 3 infection, which occurred in 31% of patients. Serious adverse reactions related to the treatment occurred in two patients: a syndrome of inappropriate antidiuretic hormone secretion and a severe gait disturbance with limb pain. Both events resolved, but MC was permanently discontinued in the first case.

**Figure 1:**
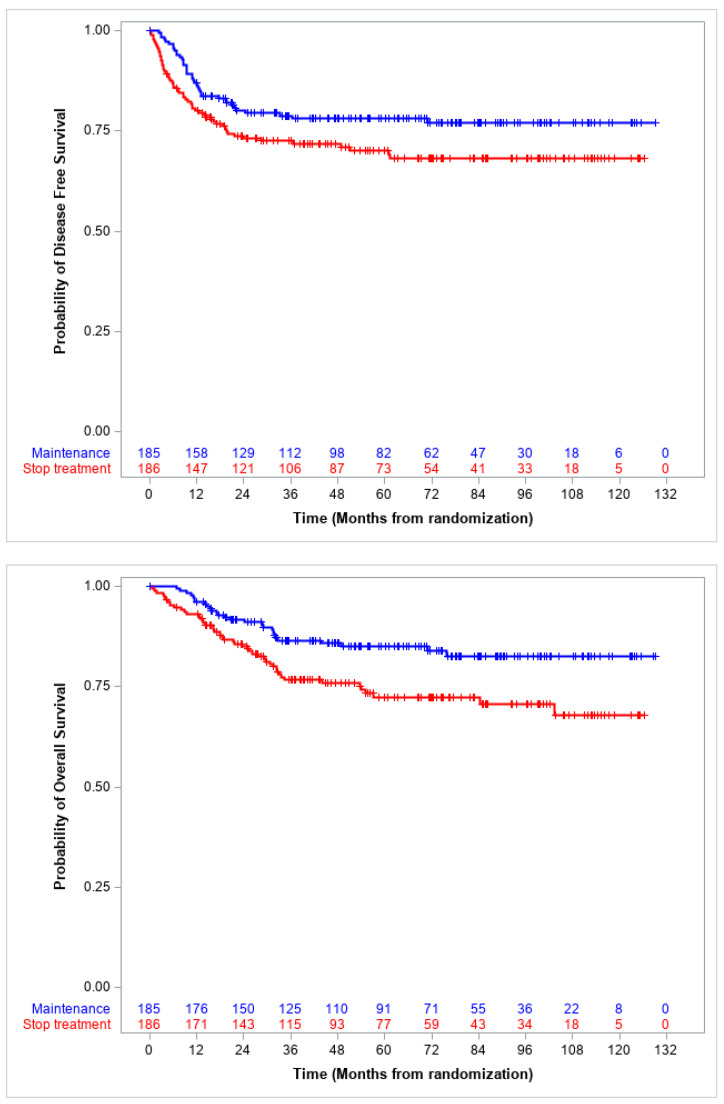
RMS 2005 trial: Disease free and overall survival by randomization arm.

In the present report, the median follow-up is 65.6 months (IQR 37.3–93.1), and the 5-year DFS in the intention-to-treat population was 70.1% (95% CI 62.6–76.3) for patients who stopped treatment, and 78.1% (95% CI 71.2–83.5) for those in the MC arm (*p* = 0.056). The corresponding 5-year OS was 72.4% (95% CI 64.6–78.7) and 85.0% (95% CI 78.5–89.6) (*p* = 0.008), respectively (Figure 1).

At the last data cut-off, 95 patients (25.3%) experienced an event and 74 died. These figures compare with 94 events and 66 deaths reported in the first publication [8].

In the EpSSG studies, MC was also added to the treatment of all patients with localized RMS and a very high risk of treatment failure or with metastasis at diagnosis, with the aim to improve the unsatisfactory results obtained in previous trials (Table 1).

Patients with localized alveolar RMS and regional nodal involvement were included in the very high-risk (VHR) group of the RMS2005 protocol. Standard chemotherapy included four cycles of IVADo (IVA plus doxorubicin) followed by five cycles of IVA. Patients in complete remission continued with MC with vinorelbine and cyclophosphamide for 24 weeks. The 5-year EFS and OS rates for VHR patients were 50.1% (95% CI, 39.8–59.5%) and 50.6% (95% CI, 39.7–60.5%), respectively These figures were better when compared with historical cohorts, but the non-randomized administration of MC made it impossible to evaluate the benefit of its addition [19]. To overcome this problem, a comparison with a similar series of patients treated without MC in COG protocols was attempted. The different strategies adopted by the two groups determined similar results and identified fusion-positive tumors as those needing innovative treatments beyond MC [30]. 

Patients aged less than 21 years with a histologic diagnosis of RMS and distant metastatic disease at diagnosis were included in the prospective international study MTS2008. Patients received the same chemotherapy described for VHR patients with IVADo/IVA in the induction phase but MC with vinorelbine and cyclophosphamide was prolonged to 48 weeks in those included in the MTS2008 protocol (Table 1). The MTS 2008 study reported a 3-year EFS of 34.9% (95% CI 29.1–40.8%) and OS of 47.9% (95% CI 41.6–53.9%). Notably, 14% of VHR and 46% of metastatic patients presented tumor progression or relapse during the induction or maintenance treatment phases [20]. This high early failure rate suggests that a more effective induction treatment is necessary for these two groups of patients and especially for those with metastases.

In the search for new active agents, the use of antiangiogenetic drugs appeared attractive on the basis of the results achieved in RMS preclinical models [31]. This gave the rationale for the use of bevacizumab in the BERNIE study, an open label multicentre phase II study born from the collaboration of the EpSSG with the Innovative Therapy for Children with Cancer (ITCC) consortium and supported by Roche. Overall, 154 patients with metastatic soft tissue sarcoma were enrolled and randomized to receive the same treatment recommended in the MTS20068 protocol (standard arm: IVADo/IVA followed by 48 weeks of MC with cyclophosphamide and vinorelbine) with the addition of bevacizumab (experimental arm) both during standard treatment (bevacizumab 7.5 mg/kg every 3 weeks on day 1 of each cycle) and maintenance phase (bevacizumab 5.0 mg/kg every 2 weeks on days 1 and 15 of each cycle). Data for the RMS group have not been reported separately but the 2-year EFS was similar in the two groups: 41% (95% CI: 28.8–52.3) in the control arm and 41% (95% CI: 29.4–53.0) in the experimental arm, showing no significant advantage from the addition of bevacizumab [21]. 

## 4. Other Experience of MC in RMS

We searched PUBMED for recent literature (January 2000 to May 2023) using the keywords rhabdomyosarcoma AND maintenance chemotherapy OR low dose chemotherapy OR metronomic (164 papers found). We also searched the references and ASCO and SIOP congresses presentations and selected publications describing clinical trials (twelve papers) excluding those published by the EpSSG (four papers) [18,19,20,21] that have been described previously. The eight selected papers are described in this section and summarized in Table 1 [22,23,24,25,26,27,28,29].

The German Cooperative Group (CWS) was the first to introduce MC in the treatment of pediatric STS and we found four papers and an abstract describing this approach [24,25,26,27,28]. The HD CWS-96 trial was open from May 1995 to December 2003 and enrolled 96 patients with metastatic RMS (74 patients) and other STS (22 patients). After standard induction therapy, children were allocated by clinician’s decision into the high-dose arm and given a cycle of thiotepa plus cyclophosphamide followed by a second cycle with melphalan plus etoposide) with stem cell rescue or in the MC arm. MC was composed of alternating cycles of trofosfamide (2 × 75 mg/m^2^/day) and idarubicine (1 × 5 mg/m^2^/day 1, 4, 7, 10) alternating with trofosfamide and etoposide (2 × 25 mg/m^2^/day). All drugs were given orally (O-TIE regimen). Each cycle was given continuously for 10 days and the interval between cycles was 3 weeks, for a total duration of 24 weeks. This was not a randomized trial, but the population in the two arms had similar characteristics at the moment of diagnosis. The study had a median follow-up of 57.4 months (range 12.3–104.2). Patients in the oral MC arm had an OS of 52% compared to 27% of those included in the high-dose arm (*p* = 0.03). The superiority of oral MC was more evident within the RMS subgroup (OS 52% vs. 15% (*p =* 0.001)) [22].

A second study focusing on patients with embryonal RMS and isolated pulmonary metastasis treated in four consecutive trials suggested an improved 5-year EFS of 75%, but in a very small subset of eight patients, when O-TIE maintenance was added to standard treatment [23].

These results prompted CWS to test MC in patients with high-risk localized STS in two consecutive trials. In the prospective non-randomized CWS2002P study, the responsible clinician had the option to add MC at the end of standard treatment for patients with high-risk and very high-risk localized RMS that had achieved a complete remission. MC included the administration of seven 3-week cycles of intravenous vinblastine 3 mg/m^2^ on days 1, 8, and 15, and oral cyclophosphamide 2 × 25 mg/m^2^/day on days 1–21, with a one week pause between the cycles. The 5-year EFS was 77% for the 155 patients who received MT versus 63% for the 49 patients who did not (*p* = 0.015); the 5-year OS was 84% versus 73% (*p* = 0.099) [24].

In the following CWS-2007 HR trial, 195 out of 337 eligible patients were randomized to stop treatment or to receive oral MC with eight 10-day courses of the O-TIE regimen already used in the HDCWS-96 trial. With a median follow-up of 4.9 years, the 3-year results were similar in the two arms. The event-free survival (EFS) in the MC arm vs. stop treatment arm was 66.2% (95% IC 57.1–76.79) vs. 75.0% (95% IC 66.8–84.3) (*p* = 0.07) and the OS 81.9% (95% IC 74.2–90.4) vs. 84.6 (95% IC 77.5–92.4) (*p* = 0.15) [25]. For more details see also Table 2.

The CWS group further tested the role of MC in patients with metastatic STS in the CWS IV-2002 and CWS DOK IV 2004 trials, where 89 patients were treated with different types of MC (Table 1). In this study, 13 patients received high-dose chemotherapy with autologous stem cells infusion and 21 patients received allogeneic bone marrow transplant. Once again, treatment allocation was at the discretion of the treating physician, prompting caution in the interpretation of the results. Patients receiving MC showed a 3-year EFS and OS of 41 and 53% (95% CI, 43 to 64%), respectively. The outcome was similar when compared to those receiving a high-dose chemotherapy regimen, but with less therapeutic burden, and significantly better when compared to those receiving an allogeneic hematopoietic stem cell transplantation [26]. 

A series of 49 patients with relapsed, refractory, or metastatic sarcoma, including 32 Ewing sarcoma, 13 RMS, and 4 other sarcomas, have been treated at the Tata Memorial Hospital in India with oral tamoxifen 40 mg/m^2^ day, divided twice daily every day, and associated with etoposide and cyclophosphamide. Both drugs were given orally at the dose of 50 mg/m^2^ for 21 days every 28 days, for a total of at least 12 cycles. Aggregated data have been reported so we can assess the effect of this type of MC in RMS. The response rate for the whole group was 59% and the median PFS was 22 months. Authors concluded this is an affordable, accessible, tolerable, and effective treatment for patients with sarcomas, especially if they live in Low and Middle-Income Countries [27]. 

A study performed in Morocco reported the use of MC in 98 patients with refractory solid tumors including 15 with RMS. The MC protocol consisted of 28-day cycles with daily oral administration of cyclophosphamide (30 mg/m^2^) from days 1 to 21, together with oral etoposide (25 mg/m^2^) from days 1 to 21, followed by a break of one week and daily valproic acid (20 mg/kg) from days 1 to 28. Treatment was very well tolerated (grade 0 or 1 toxicity was reported in 95% of cycles administered). The response rate was 14% (21.4% in RMS, but 0% in osteosarcoma) with an additional 28% of stable disease. One-year PFS was 19% but authors reported a clinical benefit in few patients [28]. 

A recent report described the use of the MC in intermediate and high-risk RMS (according to the Children Oncology Group definition that roughly corresponds to the high-risk, VHR, and metastatic patients in the EpSSG stratification system) in complete tumor remission or with PET-negative residuals at the end of standard treatment. MC was also given to patients that achieved a second tumor complete remission after a first relapse. MC was composed of the same drugs (vinorelbine and low-dose cyclophosphamide) and schedule used in the RMS2005 but differed for the duration (48 instead of 24 weeks), the dose (25–40 mg/m^2^ instead of the usual 60 mg/m^2^) and formulation (oral instead of i.v.) of vinorelbine. Despite the retrospective nature of the study, the heterogeneity of the population, and the different standard chemotherapy administered before MC, the reported 70% 3-year overall survival in non-metastatic relapsed patients is of interest [29].

## 5. Discussion

The updated results of the RMS2005 trial we present here confirm the benefit of adding six cycles of MC to the standard treatment of patients with high-risk RMS. Overall survival remains higher in the MC arm, while DFS (the primary trial endpoint), although clinically significant, still falls just short of the conventional definition of statistical significance, as in the initial publication [8]. With a moderately longer follow-up, the number of events seems to be stable. A final analysis is planned for 2024.

In our opinion, these updated results confirm the reasons that urged EpSSG to decide to change the standard treatment in high-risk localized RMS adding 6 cycles of MC with vinorelbine and low-dose oral cyclophosphamide. In light of EpSSG results, the Children Oncology Group has also amended the “intermediate risk trial” to add a similar MC regimen to standard chemotherapy with or without temsirolimus (NCT02567435). Other groups have started to adopt the same strategy [29] and this will help to obtain more information about the role of MC in RMS.

Many factors may have contributed to the efficacy of maintenance in the EpSSG study, including the type of drugs used, possible new anticancer mechanisms, and the duration of treatment.

Different mechanisms have been hypothesized to explain the activity of weekly vinorelbine and low-dose cyclophosphamide. Both drugs showed convincing evidence of activity against RMS and were not part of the multidrug regimen patients received in the first part of treatment, making the development of drug resistance against them by the tumor cells unlikely. This is one of the reasons that may explain the failure of MC on osteosarcoma as methotrexate and cyclophosphamide were used both during the standard treatment and MC [4,7]. A recent study suggested that the response rate to vinorelbine is higher in alveolar than in embryonal RMS, making this drug particularly attractive for high-risk and metastatic RMS where the alveolar subtype is more frequent [32]. However, the subgroup analysis conducted in the RMS2005 study was not able to find any specific subgroup with a significantly better outcome when treated with MC, including vinorelbine [8]. 

The concept of metronomic therapy overlaps with the definition of MC. The MC used in the RMS2005 trial cannot be defined as fully metronomic because vinorelbine was given at full doses, but cyclophosphamide was administered continuously with daily low doses. This component of MC may have acted with different mechanisms, i.e., impacting angiogenesis through endothelial cells or circulating endothelial cell killing, by inhibiting VEGF or increasing antiangiogenic molecules. In addition, the metronomic administration of cyclophosphamide seems able to restore immune function by increasing CD4+/CD8+ T cells and depletion of T regulatory cells that may play a role in favoring tumor progression and therapy resistance [14,15,16,33,34]. 

Over the years, cooperative groups tended to increase the intensity of chemotherapy and reduce its overall duration. The RMS 2005 study successfully adopted an inverse strategy, i.e., prolonging the total duration of treatment but reducing its intensity and possibly its toxicity [35]. 

The preliminary results of the CWS 2007, recently reported [25], suggest caution and seem to contradict the RMS 2005 results. CWS 2007 data need to be further specified and analyzed and possibly compared with those of the EpSSG RMS 2005 study as there are several differences between the two studies. The main characteristics of the two trials have been summarized in Table 2. In particular, the type and number of patients enrolled in the two studies appear rather different and the CWS study also included “RMS-like tumors” (i.e., undifferentiated sarcoma, extraskeletal Ewing sarcoma, and synovial sarcoma) which are known to have various levels of chemosensitivity, so results for the RMS group are awaited.

CWS2007 results seem to suggest that not all MCs are equal. It may be that the combination of trofosfamide, idarubicine, and etoposide tested in the CWS trial is not as effective as the vinorelbine-cyclophosphamide regimen. Not only are the used drugs different, but also the modality of administration. For instance, MC in the EpSSG trial included the continuous administration of one drug (cyclophosphamide) that may be important as a metronomic approach.

There are few experiences reporting the use of MC in relapsed RMS [27,28,29]. This may be another possible use of MC that needs to be explored. Low-dose chemotherapy is often used as palliative treatment, but MC may offer the possibility to consolidate a second tumor complete remission achieved after the administration of second-line chemotherapy or even after experimental therapies. 

A more general consequence of the positive result of the RMS2005 trial is the new interest in MC in other solid tumors. The possibility of prolonging the treatment in patients in complete tumor remission is under study for several tumors. As an example, in the current European Ewing sarcoma trials, the iEuroEwing and the Inter-Ewing-1 studies, the same strategy and drug combination used in RMS2005 will be randomly tested.

MC seems also an interesting concept to test the effectiveness of biological agents, such as bevacizumab or mTOR inhibitors, but too few trials have been conducted in children [21] and adults with sarcomas [3,36,37,38]. 

There are still many aspects of MC that need to be further investigated. In patients with leukemia, different drug combinations have been compared, and the interaction between drugs has been investigated as well as the best drug administration time. Treatment adherence, drug metabolism, and compliance also remain to be investigated in RMS. The possibility of using oral vinorelbine may facilitate patient compliance [29]. The problem of possible additional late effects also needs to be addressed. The addition of MC has increased the exposure of patients to higher cumulative doses of alkylating agents; this means a potentially higher risk of second tumors and fertility or endocrine impairment. Late effects correlated to vinorelbine are thought to be less relevant but still need to be investigated.

The prolongation of treatment and the risk of late effects that may be caused by MC underline the need to better identify those patients that can really benefit from this strategy. RMS2005 demonstrated that MC increases the number of cured patients, but most patients have still been successfully treated with standard chemotherapy, only as testified by the 70.1% DFS obtained in the arm that did not receive MC in the RMS2005 trial. We require minimally invasive biomarkers, able to identify the persistence of minimal amounts of disease at the end of standard therapy, so that MC could be limited to this group of patients. In this sense, the use of liquid biopsies needs to be explored [39,40]. 

## 6. Conclusions

The addition of MC to the standard treatment of patients with RMS is a recent acquisition and needs to be further explored. The ongoing EpSSG FaR-RMS study takes forward the MC vinorelbine and cyclophosphamide combination and is now answering additional questions on MC and in particular, the optimal duration, i.e., 6 months vs. 12 months in localized high-risk RMS patients and 12 months versus 24 months in very high-risk patients, meaning those with PAX fusion-positive node-positive, and metastatic disease. It is plausible that an ideal duration of MC can correlate with patient tumor burden. Moreover, the use of oral vinorelbine will be addressed, making the regimen more appreciated by patients and families by reducing the need for hospital attendance [29,41].

If it is true that many aspects of MC need to be investigated, still, its addition represents a major advancement and has changed the international standard of care for patients with RMS. 

## Figures and Tables

**Table 1:** Published trials exploring maintenance chemotherapy for patients with rhabdomyosarcoma.

Author, Year of Publication (Reference)	Study	Patients	Type of Study (No of Pts)	Maintenance Chemotherapy	Conclusion
Bisogno et al., 2019 [8]	RMS2005—international multicentre	High-risk localized RMS	Randomized (371 pts enrolled, 185 received MC)	6 cycles of i.v. vinorelbine 25 mg/m^2^ on days 1, 8, 15 and oral cyclophosphamide 25 mg/m^2^/day, on days 1 to 28.	Maintenance chemotherapy significantly increases patient OS (DFS increase was evident but not statistically significant)
Gallego et al., 2018 [19]	RMS2005—international multicentre	Very high risk localized RMS	Prospective (103 pts)	Same as RMS2005 study	The contribution of MC to OS and EFS difficult to establish. Prognostic impact of fusion status
Schoot et al., 2022 [20]	MTS2008—international multicentre	Metastatic RMS	Prospective (270 pts)	Same as RMS2005 study but longer (12 cycles)	The outcome remains poor. Not possible to determine whether the addition of MC improved the outcome in comparison with historical cohorts.
Chisholm et al., 2017 [21]	BERNIE—international multicentre	Metastatic soft tissue sarcomas	Randomized phase II	Same as in MTS2008 study with the addition of Bevacizumab in the experimental arm	The outcome was not improved by the addition of Bevacizumab
Klingebiel et al., 2008 [22]	HD CWS-96—international multicentre	Metastatic RMS	Prospective non randomized (96 pts enrolled, 51 received MC)	4 cycles of trofosfamide (2 × 75 mg/m^2^/day) and idarubicine (1x5 mg/m^2^/day 1, 4, 7, 10) alternating with 4 cyles of trofosfamide and etoposide (2 × 25 mg/m^2^/day)	Significantly superior survival for patients receiving oral MC vs. those receiving high-dose chemotherapy
Dantonello et al., 2010 [23]	CWS-91, CWS-86, CWS-91, CWS-96—international multicentre	Embryonal RMS with isolated lung metastasis	Retrospective (29 pts, 8 received MC)	Same as HD CWS-96 study	5-years EFS was significantly superior in patients receiving MC
Koscielniak et al., 2022 [24]	CWS2002P—international multicentre	High-risk localized soft tissue sarcomas	Prospective non randomized (204 pts enrolled, 155 pts received MC)	7 cycles of i.v. vinblastine 3 mg/m^2^ on days 1, 8, and 15 and oral cyclophosphamide 2× 25 mg/m^2^/day on days 1–21. One week pause between the cycles	EFS and OS were significantly superior for patients receiving MC
Koscielniak et al., 2022 [25]	CWS-2007 HR—international multicentre	High-risk localized soft tissue sarcomas	Randomized 195 enrolled, 96 received MC)	As in the HD CWS-96 study	OS and EFS were not different in the 2 groups
Tramsen et al., 2023 [26]	CWS IV-2002 and CWS DOK IV 2004—international multicentre	Metastatic RMS	Prospective non randomized (176 pts enrolled, 89 received MC)	Same as in the HD CWS-96 study (14 pts) or as in the CWS2002P study (75 pts)	MC produces better results than allogenic bone marrow transplant and similar results when compared to high-dose chemotherapy but with a less therapeutic burden
Devadas et al., 2019 [27]	Institutional study	Relapsed/refractory or metastatic sarcomas	Retrospective (13 RMS pts)	Oral tamoxifen 40 mg/m^2^/day divided twice every day, associated with etoposide and cyclophosphamide, both drugs were given orally at the dose of 50 mg/m^2^ for 21 days every 28 days, for at least 1 year.	MC is a low-cost treatment that can induce long-term remission in few patients
El Kababri M et al., 2020 [28]	Multicentre study	Refractory or relapsing solid tumors	Prospective (14 RMS pts)	cyclophosphamide (30 mg/m^2^) and etoposide (25 mg/m^2^) days 1–21, followed by a break of one week and daily valproic acid (20 mg/kg) days 1–28. All drugs were given orally	The regimen demonstrated activity against sarcoma (3 responses in RMS pts)
Lan Y et al., 2023 [29]	Institutional study	Newly diagnosed high-risk and relapsed RMS	Retrospective (57 pts)	Sam as RMS2005 study (but vinorelbine was administered orally and at a lower dose) Duration 48 weeks	Interesting results in relapsed non-metastatic pts (3-year OS 70%)

Legend: MC—Maintenance Chemotherapy; RMS—Rhabdomyosarcoma.

**Table 2:** A comparison of the main characteristics of RMS2005 and CWS2007 trials.

	RMS2005	CWS2007
Standard chemotherapy before maintenance chemotherapy	9 cycles of Ifosfamide, vincristine, actinomycin-D +/− doxorubicin	9 cycles of Ifosfamide, vincristine, actinomycin-D +/− doxorubicin
Eligible patients	High-risk localized RMS *	High-risk localized RMS * Undifferentiated sarcoma Extraskeletal Ewing sarcoma Nonresectable Synovial sarcoma
Maintenance chemotherapy	6 cycles of intravenous vinorelbine 25 mg/m^2^ on days 1, 8, 15 and daily oral cyclophosphamide 25 mg/m^2^, days 1–28	Eight 10-day courses consisting of trofosfamide (2 × 75 mg/m^2^/day) and idarubicine (×5 mg/m^2^/day 1, 4, 7, 10) alternating with trofosfamide and etoposide(2 × 25 mg/m^2^/day). All drugs were given orally
Patients randomized	371 (186 vs. 185)	195 (99 vs. 96)
Median Follow-up	60.3 months (IQR 32·4–89·4)	4.9 years (IQR 3.0–5.7)
Results **		
DFS/EFS standard arm vs. maintenance chemotherapy arm	69.8% vs. 77.6% (*p* = 0.06)	75% vs. 66.2% (*p* = 0.07)
OS standard arm vs. maintenance chemotherapy arm	73.7% vs. 86.5% (*p* = 0.009)	84.6% vs. 81.9% (*p* = 0.15)

* Both studies used the same risk stratification definition apart from patients with alveolar RMS with nodal involvement that was included in CWS2007 only ** Results: RMS2005 used 5 years DFS and OS vs. CWS2007 that used 3-year EFS and OS.

## Data Availability

Individual participant data are not publicly available since this was not foreseen by the study protocol. The protocol can be requested through the EpSSG website https://www.epssgassociation.it/en/.
